# Bioactivity of a modified human Glucagon-like peptide-1

**DOI:** 10.1371/journal.pone.0171601

**Published:** 2017-02-02

**Authors:** Fangfang Xu, Kevin Yueju Wang, Nan Wang, Gangqiang Li, Dehu Liu

**Affiliations:** 1 Biotechnology Research Institute, Chinese Academy of Agricultural Sciences, Beijing, China; 2 Department of Natural Sciences, Northeastern State University, Broken Arrow, Oklahoma, United States of America; Max Delbruck Centrum fur Molekulare Medizin Berlin Buch, GERMANY

## Abstract

Diabetes has become the third largest cause of death in humans worldwide. Therefore, effective treatment for this disease remains a critical issue. Glucagon-like peptide-1 (GLP-1) plays an important role in glucose homeostasis, and therefore represents a promising candidate to use for the treatment of diabetes. Native GLP-1, however, is quickly degraded in in the circulatory system; which limits its clinical application. In the present study, a chemically-synthesized, modified analogue of human GLP-1 (mGLP-1) was designed. Our analyses indicated that, relative to native GLP-1, mGLP-1 is more resistant to trypsin and pancreatin degradation. mGLP-1 promotes mouse pancreatic β-cell proliferation by up-regulating the expression level of *cyclin E*, *CDK2*, *Bcl-2* and down-regulating *Bax*, *p21*, and stimulates insulin secretion. An oral glucose tolerance test indicated that mGLP-1 significantly improved glucose tolerance in mice. Intraperitoneal injections of mGLP-1 into streptozotocin (STZ)-induced type 2 diabetic mice significantly reduced blood sugar levels and stimulated insulin secretion. Oral gavages of mGLP-1 in diabetic mice did not result in significant hypoglycemic activity.

## Introduction

Diabetes is a major chronic, systemic metabolic disease that threatens human health and reduces the quality of life [1]. In 2015, the International Diabetes Federation (IDF) reported that 415 million adults have diabetes worldwide and that by 2040, approximately 10% of the world’s population will be living with this disease [2]. Roughly 90% of all cases of diabetes in humans belong to type 2 diabetes, which is characterized by β-cell dysfunction and insulin resistance [3]. Both of these parameters impair insulin secretion, induce a glucose metabolism disorder, and thus lead to continuous hyperglycemia. Long-term hyperglycemia causes dysfunction and damage to various organs; especially the eyes, kidneys, heart, blood vessels, and nerves.

β-cell dysfunction resulting from glucose toxicity is potentially reversible if metabolic control can be restored [4]. Therefore, controlling and reducing the glucose toxicity may delay the deterioration of β-cell function. At the present time, drugs that effectively control blood sugar levels are not available. Glucagon-like Peptide-1 (GLP-1), a short incretin hormone, is primarily synthesized and secreted by proximal small intestine enteroendocrine L-cells [5]. The mature form of GLP-1 is comprised of 30 amino acids and GLP-1 has pleiotropic therapeutic effects on β-cell restoration and proliferation [6–8]. GLP-1 stimulates insulin secretion in response to glucose, lipids, or mixed oral nutrient ingestion in a glucose dependent manner and becomes inactive when the concentration of blood sugar is less than 3.6 mM. Hence, GLP-1 is an excellent candidate for use as a prophylactic treatment for maintaining glucose homeostasis in diabetic patients and has been extensively investigated. The very short plasma half-life (less than 2 minutes in the circulatory system), however, limits its pharmacotherapy study and clinical application [9–11]. Dipeptidyl peptidase IV (DPP-IV)-mediated cleavage at Ala^8^ of mature N-terminal GLP-1 results in the production of inactivated N-terminal truncated peptides, GLP-1(9–36) or GLP-1 (9–37). In addition, the kidney also quickly eliminates GLP-1 [12].

Various approaches to extend the half-life of GLP-1 have been investigated, such as developing GLP-1 analogues and DPP-IV inhibitors. DPP-IV- mediated GLP-1 degradation can be avoided by identifying naturally existing DPP-IV-resistant GLP-1 analogs, such as exendin-4 [13], by making N-terminal modifications of GLP-1 [14] or shielding of its cleavage site [15]. In this study, a trypsin-resistant GLP-1 analogue (mGLP-1) was designed and synthesized. The Ala^8^, Lys^26, 34^ amino acid residues in human GLP-1 (7–36) were replaced with Gly^8^, Gln^26^, and Asp^34^, respectively. Arg^36^ was retained at the C-terminal in order to maintain bioactivity. The mGLP-1 analogue exhibited a significant level of resistance to trypsin-induced cleavage. The bioactivity of mGLP-1, both *in vitro* and *in vivo*, was also investigated. Results indicated that mGLP-1 promoted pancreatic β-cell proliferation, stimulated insulin secretion, and lowered glucose levels in mice.

## Materials and methods

### Peptides and experimental mice

Modified human GLP-1 (mGLP-1) and native human GLP-1were both synthesized by ChinaPeptides Co., Ltd. (Shanghai, China). A total of thirty-eight week-old, SPF grade, male KM mice were purchased from Vital River Laboratories Company (Beijing, China) and maintained with standard food pellets under a 12 h light-dark cycle. All animal protocols were reviewed and approved by the Chinese Academy of Agriculture Sciences (CAAS) Institutional Animal Ethical and Welfare Committee (No: BRISPF-2016-03). All mice (30) were euthanized three months after experiments by CO_2_ asphyxiation.

### Trypsin and pepsin proteolysis assay

The resistance of GLP-1 and mGLP-1 to trypsin proteolysis was evaluated by incubating samples of mGLP-1 (10 mg/mL) or GLP-1 (10 mg/mL) with trypsin (4 mg/mL) in PBS (pH 7.4) buffer (1×) at 37°С for 1, 2, 4, 8, 12, 24, 48-h or 1, 2, 4, and 8-h, respectively. GLP-1 (2.5 mg/mL) or mGLP-1 (2.5 mg/mL) were also incubated with 1.0 mg/mL pancreatin (TCI, JPN) in PBS (pH 7.4) at 37°С over various time intervals to evaluate the stability against simulated intestinal fluid. Resistance to pepsin degradation was evaluated by incubating mGLP-1 (1 mg/mL) or GLP-1 (1 mg/mL) with pepsin (2 mg/mL, Sigma) in a hydrochloric acid (HCl) solution (pH 1.5~2.0) at 37°С for 10min. Samples (20 μL) treated with either trypsin or pepsin for different lengths of time were then mixed with SDS-PAGE loading buffer (2×), heated at 100°С for 10 min, and then analyzed with SDS-PAGE. Protein bands on the gels were visualized by silver staining.

### Cell proliferation

Mouse insulinoma cells (MIN6) (iCell Bioscience, Inc. Shanghai, China) were cultured in a 25 cm^2^ cell culture flasks and then seeded into 96-well microplates at a density of 6.0×10^4^ cells per well for the cell proliferation assay. The culture was incubated in an incubator with 5% CO_2_ at 37°С and 95% RH for 12 h. mGLP-1 or GLP-1 at different concentrations (0.3, 3, 15, and 30-μM) was then mixed into the cell cultures. After 48 h of incubation, 10 μL CCK-8 solution (cell counting kit, Dojindo China Co., Ltd. Shanghai, China) was gently mixed into the culture and the culture was further incubated for 1 to 4 hours. A microplate reader (Bio-Rad, USA) was used to measure cell viability at a 450 nm wavelength (OD450). The cell viability ratio (CRV) was calculated as (A-A_0_)/A_0_ × 100% (where A was the absorbance of the treated cell culture and A_0_ was the OD450 value of a blank (RPMI 1640 medium only)).

### RT-qPCR of mGLP-1-regulated gene expression

For screening the expression level of cell proliferation and apoptosis-related genes, total RNA from (GLP-1-treated, mGLP-1-treated, and control) MIN6 cells was extracted using a MiniBEST Universal RNA Extraction Kit (Takara) according to the manufacturer’s instructions. The total RNA in each sample was quantified by UV spectroscopy. Generation of cDNA was performed using a PrimeScript™ RT reagent Kit (Takara) according to the manufacturer’s instructions. RT-qPCR was performed to detect the levels of *Bcl-2*, *Bax*, *cyclin E*, *CDK2*, and *p21* genes expression. The gene-specific primers used in the analysis are listed in Table 1. RT-qPCR analysis was performed using a standard SYBR Fast qPCR Mix kit (Takara) protocol on a 7900HT Fast Real-Time PCR System (Applied Biosystems). Relative expression levels were calculated using the 2^-ΔΔCt^ method.

**Table 1:** Gene-specific primers designed for the designated mouse genes (Genbank ID) and used to determine the expression levels by RT-qPCR.

Name	Genbank ID	Sequence (5'->3')	product length (bp)
GAPDH	gi|53237094|	TTGCAGTGGCAAAGTGGAGA	175
		GTCTCGCTCCTGGAAGATGG	
Bcl-2	gi|929981607|	GAACTGGGGGAGGATTGTGG	176
		CCAGACATGCACCTACCCAG	
Bax	gi|133778943|	CACTAAAGTGCCCGAGCTGA	167
		TGAGGACTCCAGCCACAAAG	
Cyclin E	gi|443939|	TATGGTGTCCTCGCTGCTTC	202
		GGGTCTGGATGTTGTGGGAG	
CDK2	gi|162287299|	GTGGTACCGAGCACCTGAAA	188
		CTGGCCAAACCACCTCATCT	
p21	gi|50300976|	TTGCACTCTGGTGTCTGAGC	190
		GGGAAGGGCTGGAATGTTCT	

### Insulin secretion assay

Three days after incubation in an incubator with 5% CO_2_ at 37°С and 95% RH, MIN6 cells were washed two times using Dulbecco's phosphate buffered saline (DPBS) plus 0.1% BSA (Sigma), and then kept in starvation in DPBS with 0.1% BSA for an hour. GLP-1 (15 μM) or mGLP-1 (15 μM) was then added along with 10mM glucose (10 mM) for various lengths of time as described by Brandsma [16]. Cell culture supernatant was then collected and insulin was measured with a mouse insulin ELISA kit (JiNingShiYe Ltd. Shanghai, China) according to the manufacturer’s instructions.

### Oral Glucose Tolerance Test (OGTT) in mice

OGTT was conducted on eight-week-old mice that had fasted overnight. A bolus of glucose (2 g/kg body weight) was loaded into the stomach via oral gavage. Then, 30 min or 90 min prior to the OGTT, mGLP-1, GLP-1, or PBS alone was delivered into the mice by intraperitoneal (IP) injection at a concentration of 0.1 mg/kg body weight. The glucose level in blood samples obtained from tail veins was measured at 0, 15, 30, 60, and 120-min after gavage with a glucometer (Sannuo, Changsha, China) as described by Yuelin Kong et al [17].

### Glucose levels and insulin secretion in STZ-induced mice

A diabetic condition was induced in eight-week-old mice by intraperitoneal injection (IP) of STZ (30 mg/kg body weight, Beijing BioDee Biotechnology Co., Ltd.) for 4 consecutive days after being fed a high diet food (with crude protein content ≥15%, crude fat ≥12%, and about 4000 kcal/kg calories, data provided by Beijing Vital River Laboratory Animal Technology Co., Ltd, Beijing, China) for 1 month [18]. GLP-1 or mGLP-1 (2 mg/kg body weight) was administered by IP injection. PBS alone was injected as a negative control. Glucose levels were measured at multiple time points after injection in blood samples obtained from tail veins. Insulin concentration at the 180 min time point after injection, when blood sugar levels were at their peak, was measured with a mouse insulin ELISA Kit (JiNingShiYe Ltd. Shanghai, China).

### Statistical analysis

All data are expressed as a mean ± standard deviation (SD), and significant differences between treatments were determined by ANOVA using IBM SPSS Statistic 19.0 software. A value of *p*< 0.05 (*) was considered as statistically significant.

## Results and discussion

### Design and synthesis of mGLP-1

GLP-1 is a potent incretin hormone which is composed of 30 amino acids. It plays a critical role in glucose homeostasis via glucose-dependent insulin production and secretion, restoration of impaired β-cell secretory function and inhibition of glucagon secretion, etc. The multiple roles of GLP-1 on the process of glucose-stimulated insulin secretion makes it an ideal candidate for the treatment of type 2 diabetes. Its short half-life (less than 2 min), however, significantly limits its clinical application.

**Fig 1:**
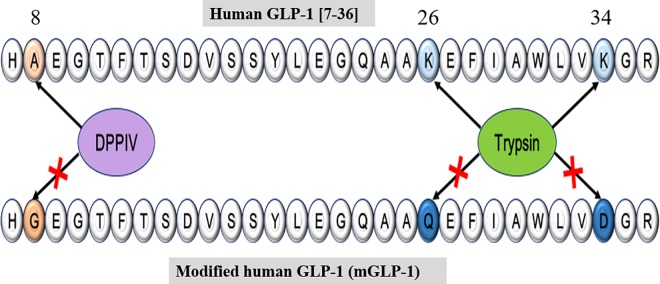
Design of modified mature human GLP-1 (mGLP-1). The amino acid sequence of human native GLP-1 (above) and the synthesized, modified mGLP-1 (below). Substituted amino acids are indicated in color: Ala^8^→Gly^8^, Lys^26, 34^→Gln^26^, Asp^34^. The blue substitutions were designed to make the mGLP-1 resistant to trypsin degradation and the brown substitution was designed to inhibit DPPIV degradation.

In the present study, a functional, long-acting, protease-resistant mGLP-1 analogue was developed by making several amino acid substitutions in native GLP-1 (Fig 1). When GLP-1 is secreted in response to the assimilation of nutrients, the ubiquitous enzyme, DPP-IV, immediately cleaves the mature GLP-1 peptide at His^7^-Ala^8^ and produces two inactive forms, GLP-1(9–36) amide and GLP-1(9–37). GLP-1(9–36) amide may even act as an antagonist on the pancreatic receptor [19]. DPP-IV cleavage is the major factor responsible for the rapid degradation of GLP-1. When Ala^8^ is replaced by Gly^8^, as in the potent GLP-1 receptor agonist exdendin-4 [20], it becomes resistant to DPP-IV-mediated degradation. Lys residues are susceptible to serine protease cleavage. In previous studies, when Lys^26, 34^ was replaced with Gln^26^ and Asp^34^, the modified GLP-1 also exhibited greater resistance to proteolytic digestion than the native peptide [14, 21]. In order to eliminate the DPP-IV target in GLP-1 and increase protease resistance, Ala^8^ was replaced with Gly^8^, and Lys^26, 34^ was replaced with Gln^26^ and Asp^34^ to synthesize a modified human GLP-1 (mGLP-1). The computed parameters of ExPASy—ProtParam (http://web.expasy.org/protparam) estimated the half-life of mGLP-1 and GLP-1 are 3.5 hours in mammalian reticulocytes and over 10 hours in *Escherichia coli*, respectively. The instability index (II) of mGLP-1 is computed to be 16.92 which classifies the protein as stable.

### mGLP-1 is resistant to trypsin and pancreatin degradation but not pepsin hydrolysis

**Fig 2:**
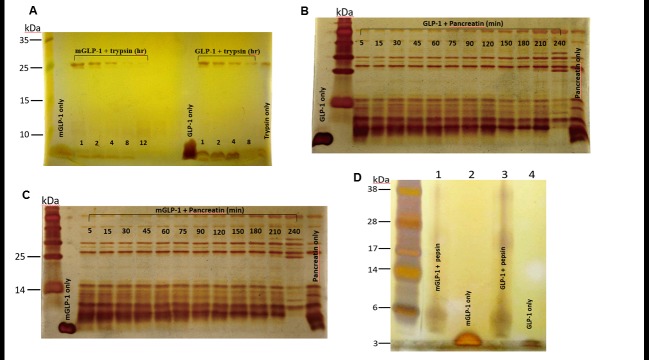
Resistance to trypsin cleavage, pancreatin degradation and pepsin hydrolysis. Both GLP-1 and mGLP-1 were treated with trypsin (**A**), pancreatin (Band C) or pepsin (**D**) at 37°C. The reaction solutions were sampled at different time points and analyzed by SDS-PAGE. Protein bands were visualized using silver staining. mGLP-1 only; mGLP-1 incubated with trypsin for 1, 2, 4, 8, and 12 hours, respectively; GLP-1 only; GLP-1 incubated with trypsin for 1, 2, 4, and 8 hours, respectively. Negative control is trypsin only. GLP-1 (**B**) and mGLP-1 (**C**) were incubated with pancreatin for 5, 15, 30, 45, 60, 75, 90, 120, 150, 180, 210, and 240-min, respectively. (**D)** Lane 1: mGLP-1 incubated with pepsin; Lane 2: mGLP-1 only; Lane 3: GLP-1 incubated with pepsin; Lane 4: GLP-1 only.

Although GLP-1 is susceptible to proteolytic enzymes, our results, utilizing silver-stained electrophoretic gels, indicated that mGLP-1 was more resistant than native GLP-1 to trypsin and pancreatin cleavage but not pepsin degradation (Fig 2). mGLP-1 was resistant to trypsin cleavage for up to 8 hours, whereas native GLP-1 was completely degraded at 8-h (Fig 2A). Both mGLP-1 and GLP-1 were quickly degraded after 5 min with pancreatin reaction (Fig 2B and 2C). After 15 min in pancreatin, GLP-1 was completely destroyed (Fig 2B), whereas faint mGLP-1 was still detectable (Fig 2C). These results demonstrate that replacement of Lys^26, 34^ with Gln^26^, and Asp^34^ in mGLP-1 result in increased resistance to trypsin and pancreatin digestion.

In contrast to trypsin, silver staining of electrophoretic gels indicated that GLP-1 and mGLP-1 were both degraded by pepsin within 10 min (Fig 2D). Neither native GLP-1 nor mGLP-1 was resistant to pepsin degradation. This is because pepsin mainly hydrolyzes peptide bonds of aromatic amino acids and acidic amino acids [22]. The presence of those bonds in GLP-1 and mGLP-1 increase their susceptibility to pepsin hydrolysis.

### mGLP-1 promotes mouse pancreatic β-cell proliferation, regulates gene expression, and stimulates insulin secretion

**Fig 3:**
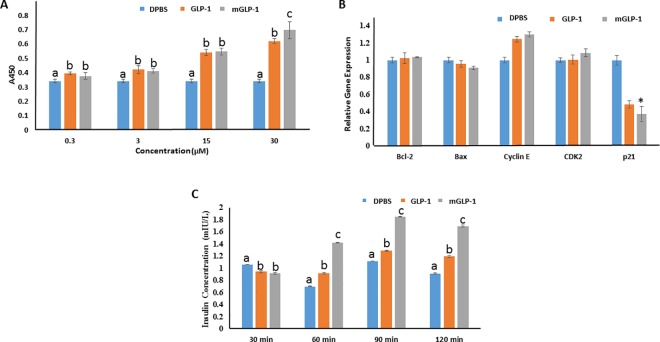
**Mouse pancreatic β cell proliferation (A), RT-qPCR analysis of gene expression (B), and insulin secretion (C)**. MIN6 cells were seeded into flat-bottomed 96-well microtiter plates at a density of 6.0×10^4^ cells per well. (**A**) Cell proliferation assay: cells were treated with various concentrations (0.3, 3, 15 and 30 μM) of mGLP-1 or GLP-1 for 48h, 10 μL CCK8 was then added to each well and incubated for 1 to 4 hours to determine the optimal reading of OD_450_. The control was cell cultures treated with DPBS only. (**B**) Relative expression of genes related with proliferation and apoptosis in cells treated with mGLP-1, GLP-1, or DPBS as determined by RT-qPCR (n = 3). (**C**) Insulin secretion assay: Mouse pancreatic β cells were incubated with 15 μM GLP-1 or mGLP-1 in the presence of glucose (10 mM) for 30, 60, 90, or 120 min. The controls were cells incubated with just DPBS in the presence of 10mM glucose. Cell culture supernatants were collected and insulin concentration determined using a mouse insulin ELISA kit (JiNingShiYe Ltd. Shanghai, China). Data shown represent the mean ±SD (n = 6). Different letters or (*) indicate significant difference at *p*<0.05.

Insulin, which is the most potent anabolic human hormone known to decrease glucose levels, is produced and released by β-cells in response to elevated levels of blood glucose [23, 24]. The impairment of β-cells results in insulin deficiency, which in turn promotes elevated blood glucose levels. A chronically elevated blood glucose level is toxic to β-cells, causing even greater impairment and damage to β-cells [23]. GLP-1 has multiple beneficial effects on β-cells, including increasing the number of β-cells by inhibiting apoptosis, enhancing β-cell neogenesis, and promoting their proliferation [25, 26]. In the present study, the mouse pancreatic β-cell line, MIN6, was used to determine the effect of mGLP-1 on cell proliferation (Fig 3A and 3B). Both mGLP-1 and GLP-1 stimulated MIN6 cells proliferation in a dose-dependent manner. At a concentration of 30 μM, mGLP-1 exhibited greater cell viability rate (104.10% ± 1.94%) than GLP-1 (81.37% ± 5.87%) (P≤0.05) (Table 2). These results indicate that mGLP-1 performed in a similar manner to native human GLP-1 in promoting MIN6 β-cell proliferation. Relative to GLP-1, mGLP-1 was more stable when incubated with MIN6 cells for 48 hours *in* vitro and induced a higher rate of cell proliferation. The relative expression level of genes related to cell proliferation and apoptosis was also consistent with the effect of GLP-1 and mGLP-1 on the level of cell proliferation (Fig 3B). The cyclin E-CDK2 complex plays an important role in regulating the G1 phase of the G1/S cell cycle [27], while p21 is a universal cyclin-dependent kinase inhibitor (CKI) [28]. The relative expression of *cyclin E* and *CDK2* genes were upregulated in mGLP-1- and GLP-1-treated cells, while the *p21*gene was down-regulated. *Bcl-2* and *Bax* genes are two important members of the *Bcl-2* gene family but they have the opposite function of either inhibiting or promoting cell apoptosis, respectively [29]. The relative expression of *Bcl-2* was slightly upregulated in GLP-1- and mGLP-1-treated cells while *Bax* was down-regulated.

**Table 2:** Cell viability ratio (CRV) of MIN6 cells treated with different concentrations of GLP-1 or mGLP-1.

Concentration (μM)	CRV (%)	
	mGLP-1	GLP-1
0.3	10.58	15.65
3	20.58	23.65
15	60.26	58.56
30	104.10	81.37

GLP-1 stimulates pancreatic β-cells insulin secretion in a glucose-dependent manner. Therefore, the ability of mGLP-1to stimulate insulin secretion in a mouse pancreatic β-cell line, MIN6, was assessed. In this assay, 15μM GLP-1 or mGLP-1, in conjunction with 10 mM glucose, was incubated with MIN6 cells for various times. As illustrated in Fig 3C, both GLP-1 and mGLP-1 promoted insulin release from MIN6 cells, relative to 10 mM glucose alone. It appeared that mGLP-1 had a greater ability than GLP-1 to stimulate insulin secretion. At 60, 90 and 120-min of incubation, mGLP-1 clearly induced a higher level of insulin secretion from MIN6 cells than did GLP-1. Furthermore, the relative gene expression level of pancreatic-specific and duodenal homeobox gene 1 (*Pdx1*) and *Ins1* were significantly up-regulated in mGLP-1- and GLP-1-treated cells (data not shown). Many transcription factors are involved in regulating the expression of the insulin gene and studies have linked the expression of the Pdx1 transcription factor to the occurrence of type 2 diabetes [30]. Pdx1, as a transcription factor expressed in pancreatic endocrine cells, promotes the expression of the pro-insulin gene, *Glut2* gene, as well as other genes [31]. Thus far, the collective results indicate that mGLP-1 retains the bioactivity of native GLP-1 and is more stable and potent than GLP-1 *in vitro*.

### mGLP-1 improves Oral Glucose Tolerance (OGTT) in mice

**Fig 4:**
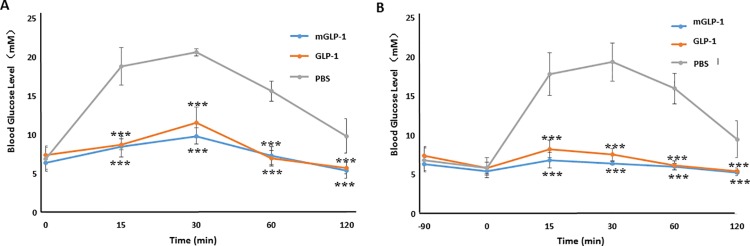
Oral glucose tolerance test (OGTT) in mice. GLP-1 or mGLP-1 was administered to mice by intraperitoneal injection (IP) 30min (**A**) or 90 min (**B**) prior to a glucose challenge (OGTT). Blood glucose level was analyzed with a glucometer (Sannuo, Changsha, China). Data shown represent the mean ±SD (n = 6). *** indicates a significant difference at *p*<0.001.

An OGTT test was performed in order to assess the bioactivity of mGLP-1 on blood glucose (Fig 4). The glucose level in the plasma of mice was significantly (p<0.001) reduced at various time points (15, 30, 60 and 90-min) in mice that received a bolus administration of glucose after receiving a prior intraperitoneal injection of GLP-1 or mGLP-1. The level of glucose reduction in mice treated with mGLP-1 or GLP-1 30 min prior to a glucose challenge was not significantly different (Fig 4A). mGLP-1 and GLP-1 also decreased blood glucose significantly when they were administered 90 min prior to the bolus administration of glucose (Fig 4B). These data indicate that mGLP-1 improved glucose tolerance and promoted insulin secretion in mice in a manner similar to native GLP-1.

### mGLP-1 lowers plasma glucose in STZ-induced type 2 diabetic mice

**Fig 5:**
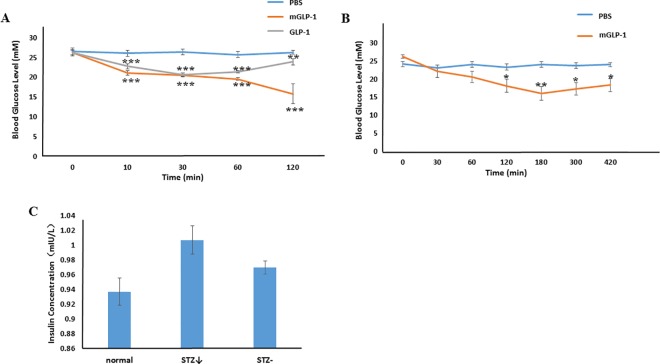
Glucose levels and insulin secretion in STZ-induced mice. Blood glucose levels in measured in STS-induced mice that received an intraperitoneal injection (IP) of PBS (control), GLP-1 or mGLP-1. (**A**) The blood glucose level was measured at 0, 10, 30, 60 and 120 min. (**B**) Intraperitoneal injection (IP) of PBS (control) or mGLP-1 in STZ-induced diabetic mice. The blood glucose level was measured at 0, 30, 60, 120, 180, 300, and 420 min. **(C)** Insulin secretion at the 180 min time point (n = 3). **STZ↓**: mGLP-1-treated STZ-induced diabetic mice with reduced blood sugar; **STZ-**: STZ-induced diabetic mice with high glucose level. Data shown represent the mean ±SD (n = 6, except where noted). * represents *p*<0.05; ** represents *p*<0.01; *** represents *p*<0.001.

STZ is toxic to pancreatic β-cells and is often used to experimentally induce diabetes mellitus in mouse and rat. As compared to intraperitoneal injection of phosphate-buffered saline (PBS) alone, injection of mGLP-1 or GLP-1 into STZ-induced diabetic mice both significantly decreased blood glucose levels over a 10–120 min period of time (Fig 5A). Glucose levels in GLP-1-treated mice initially decreased relative to the control within the first 30 min but then gradually increased from 30 to 120 min. In contrast, blood glucose levels continually decreased in mGLP-1-treated mice over the entire 120 min (Fig 5A). In fact, glucose levels continually decreased for up to 180 min in mGLP-1-treated mice, after which time they gradually increased from 180–420 min. However, glucose levels at the latest time point were still lower than in PBS-treated mice (Fig 5B). In comparison, glucose levels were significantly lower in mGLP-1-treated mice for 300 min longer than in GLP-1-treated mice. These results demonstrate that GLP-1 has less potency than mGLP-1 in lowering blood glucose levels. This may be due to the shorter half-life of native GLP-1 caused by DPP-IV, trypsin, or pancreatin digestion as illustrated in Fig 2. mGLP-1 may be more resistant to DPP-IV degradation than GLP-1 due to the mutation of the DPP-IV target amino acid and the ability of mGLP-1 to stimulate the release of insulin as shown in Fig 5C. Therefore, mGLP-1 may have a longer half-life than GLP-1 and exhibit prolonged longer period of biological activity than native GLP-1.

It is interesting to note that when an oral gavage was used to administer either GLP-1 or mGLP-1 to STZ-induced diabetic mice, no differences in glucose levels were observed at various time points relative to PBS-treated mice (data not shown). Small peptides, such as GLP-1 or mGLP-1, can be easily degraded in the alimentary canal due to the presence of various proteases, such as pepsin, a powerful proteolytic enzyme [32]. It is also possible that native GLP-1 and mGLP-1 are metabolized by the liver and therefore, do not have a chance to be systemically distributed in the blood stream [33]. Peptides and larger proteins have become increasingly explored as therapeutic agents because of their efficacy, potency and selectivity [34]. Oral administration of drugs is the most widely-used, and preferred method of delivering therapeutic agents. Therefore, further studies will be required to enhance the absorption of the mGLP-1 analogue and improve its resistance to degradation by proteases.

In summary, the present study demonstrated that synthetic mGLP-1 is more resistant to trypsin and pancreatin than native GLP-1. mGLP-1 promotes β-cell proliferation by increasing the relative expression of *cyclin E*, *CDK2*, *Bcl-2* genes and down-regulation of the *p21* and *Bax* genes; all of which are involved in the regulation of cell proliferation and apoptosis. mGLP-1 also improved glucose tolerance in mice. Collectively, the data in the present study demonstrates that mGLP-1 significantly reduces blood sugar levels and stimulates insulin secretion in mice. Further research and development of mGLP-1 will increase its potential as a therapeutic agent to be used as a GLP-1 analogue in type 2 diabetic patients.
